# The Efficacy of Lidocaine in Laryngospasm Prevention in Pediatric Surgery: a Network Meta-analysis

**DOI:** 10.1038/srep32308

**Published:** 2016-09-02

**Authors:** Xiaojing Qi, Zhoupeng Lai, Si Li, Xiaochen Liu, Zhongxing Wang, Wulin Tan

**Affiliations:** 1 Department of Anesthesiology, The First Affiliated Hospital, Sun Yat-sen University, Guangzhou 510080, China; 2 Zhongshan School of Medicine, Sun Yat-sen University, Guangzhou 510080, China

## Abstract

Higher incidence and worse outcomes of laryngospasm during general anesthesia in children than adults have been reported for many years, but few prevention measures are put forward. Efficacy of lidocaine in laryngospasm prevention has been argued for many years and we decided to design this network meta-analysis to assess the efficacy of lidocaine. We conducted an electronic search of six sources and finally included 12 Randomized Controlled Trials including 1416 patients. A direct comparison between lidocaine and placebo revealed lidocaine had the effect on preventing laryngospasm in pediatric surgery (RR = 0.46, 95% CI = [0.30, 0.70], P = 0.0002, I^2^ = 0%). Both subgroup analysis and network analysis demonstrated that both intravenous lidocaine (subgroup: RR = 0.39, 95% CI = [0.18, 0.86], P = 0.02, I^2^ = 38%; network: RR = 0.25, 95% CI = [0.04, 0.86]) and topical lidocaine (subgroup: RR = 0.37, 95% CI = [0.19, 0.72], P = 0.003, I^2^ = 0%; network: RR = 0.14, 95% CI = [0.02, 0.55]) was effective in laryngospasm prevention, while no statistical difference was found in a comparison between intravenous and topical lidocaine. In conclusion, both intravenous and topical lidocaine are effective in laryngospasm prevention in pediatric surgery, while a comparison between them needs more evidences.

The concept of laryngospasm prevention during general anesthesia was investigated quite intensively in recent years. Laryngospasm duiring general anesthesia leads to emergency in which the patient develops obstruction of the upper airway and thus causes oxygen desaturation and even death, and this could be worse in children^1^. Many factors including the intubation of ventilation device, injures and inhaled anesthetic stimulate the pharynx, larynx and upper trachea, causes lots of perioperative events including laryngospasm, which is commonly seen in pediatric anesthesia, and most frequently occurred during the immediate postinduction period (75.5%) compared with extubation or during recovery^2^. Anesthetists have been administering lidocaine intravenously or topically to prevent perioperative events during pediatric general anesthesia for many years. However, some studies have proposed that lidocaine prevents perioperative events such as cough and agitation but not laryngospasm^3^,^4^,^5^,^6^. Given that most studies are underpowered due to low incidence of laryngospasm or small-sized candidates, we decided to conduct a network meta-analysis of randomized controlled trials that evaluated the effect of intravenous or topical lidocaine in preventing laryngospasm during pediatric general anesthesia.

## Methods

### Literature search

Potentially eligible articles were obtained through searching the PubMed, Scopus, Web of Science, ScienceDirect and the Cochrane Central Register of Controlled Trials (CENTRAL) on 18 October 2015 and again on 16 March 2016, and we also conducted a search of clinicaltrials.gov. The key subjects used in literature search in PubMed were (laryngismus[All Fields] OR laryngospasm[All Fields] OR laryngospasms[All Fields] OR “laryngeal spasm” [All Fields] OR “laryngeal spasms” [All Fields] OR emergence [All Fields]) AND (lidocaine[All Fields] OR lignocaine[All Fields] OR xylocaine[All Fields]). We also scanned the references of relevant reviews and original articles to find additional citations of interest.

### Eligibility

Two researchers (XJQ and ZPL) independently assessed the suitability of the identified articles by scanning the title and abstract. Then we read the full text of those articles, which were chosen by at least one researcher, to evaluate whether they met the inclusion criteria. All uncertainties over eligibility were solved through discussion and a third researcher arbitrated when no agreement reached.

### Inclusion criteria

The included studies must meet two basic criteria. First, it compared lidocaine with a control or different routes of administration of lidocaine. Second, it reported the incidence of laryngospasm.

### Exclusion criteria

We excluded studies in adults or animals and non-RCT studies. We also excluded those studies that we could not get a full-text form for we could not assess their quality. Non-English articles were not excluded.

### Data retrieval

Two authors (XJQ and ZPL) independently extracted data from each qualified article according to a self-designed data collection form in Excel and then cross-checked the results, including the following information: (1) Source; (2) ASA physical status; (3) Age; (4) Surgery; (5) Anesthetic technique; (6) Type of airway device; (7) Intervention of the experimental group and the control group; (8) Dose of lidocaine; (9) Timing of administration; (10) URTI (upper respiratory tract infection) or not; (11) Number of laryngospasm in different groups; (12) Sample size of different groups. Discrepancy was settled by discussion between the two authors. And a third author (W.T.) arbitrate when no consensus could be reached.

In our meta-analysis, we defined laryngospasm as a condition characterized by stridor, partial or total occlusion of cords, no airflow exchange or cyanosis but not cough, hoarseness or bronchospasm. When laryngospasm was reported in classified severity, we extracted the data from the targeted category. If laryngospasm was not defined clearly or reported together with bronchospasm, we contacted the lead author for more information. When we were unable to find a contact information or obtain more detailed data, we extracted the undefined or mixed data directly. Besides, the blinding of outcome assessment could also affect the results. So we conducted a meta-regression analysis to confirm whether the undefined or mixed data and the blinding of outcome assessment mattered to the pooled results.

We also included studies written in languages other than English and Chinese, then we turned to a translator for help.

### Statistics

We assessed the risk of bias in the included RCTs using the Cochrane Handbook for Systematic Reviews of Interventions^7^. Two authors (XJQ and ZPL) independently assessed the risk of bias and then cross-checked the results. Differences were resolved by discussion between the two authors. When no consensus could be reached, a third author (WLT) arbitrate.

We used risk ratio (RR) to summarize the dichotomous data, and considered the statistical difference to be significant when the 95% CI didn’t cover the value of 1.

We analyzed the data using the Review Manager (version 5.2) in both random and fixed effect model and presented the results in forest plots in direct comparisons between lidocaine and placebo as well as between every two different subgroups. Heterogeneity was assessed by the I^2^ statistic. We changed different models and excluded the included RCTs one by one for sensitivity analysis. Small study effects, including publication bias, were assessed using a contour-enhanced funnel plot combined with trim and fill in Stata (version 12.0)^8^. A meta-regression analysis was conducted using Stata (version 12.0) including the following covariates: type of surgery (tonsillectomy or adenoidectomy vs. others), Anesthetic gas (isoflurane vs. others), airway device (tracheal tube vs. laryngeal mask airway), route of administration (intravenous vs. topical), timing of administration (before extubation vs. before intubation), definition of laryngospasm (undefined vs. mixed vs. accurate) and blinding of outcome assessment (low risk vs. unclear and high risk).

In network comparisons, we used Addis (version 1.16.5) to help us compare the effect of different laryngospasm interventions (topical lidocaine, intravenous lidocaine and placebo) in both consistency and inconsistency model, and R software (version 3.03) to draw an evidence network. Rank probabilities was measured by Bayesian probability analysis. And evaluation of inconsistency was carried out by using the node-splitting analysis.

## Results

**Figure 1:**
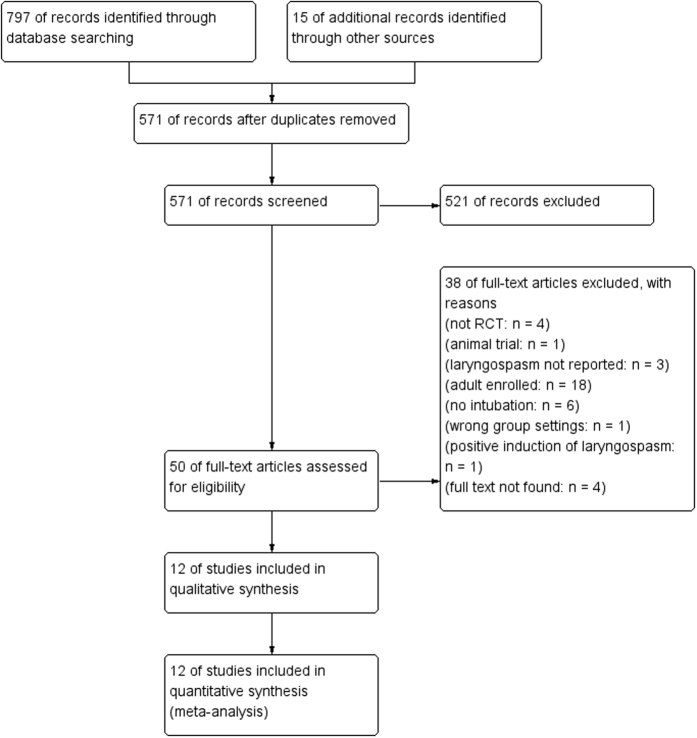
Flow diagram depicting the stages of the meta-analysis. The number of studies (n) identified, screened, excluded, and included are detailed.

A total of 571 records were identified through database searching and other sources, the full texts of 50 articles were examined in detail. And finally we included 12 RCTs^3^,^4^,^5^,^9^,^10^,^11^,^12^,^13^,^14^,^15^,^16^,^17^ with a total of 1416 participants. Searching process was shown in the flowchart (Fig. 1). Of the included studies, ten were available in English, one in Chinese and one in Korean.

**Figure 2:**
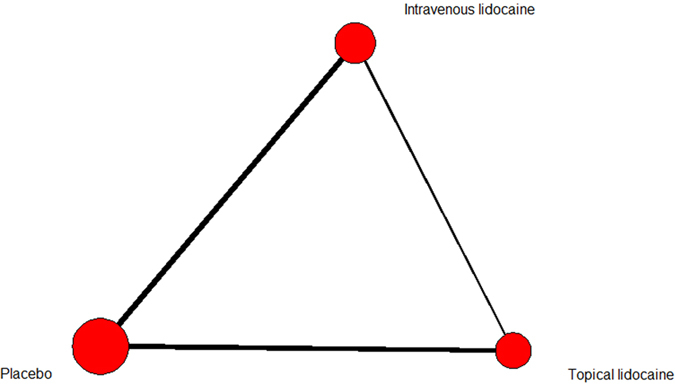
Network of lidocaine usage in laryngospasm prevention. The size of treatment nodes (red circles) reflected the number of studies. The thickness of lines represented the number of trials in that comparison.

**Figure 3:**
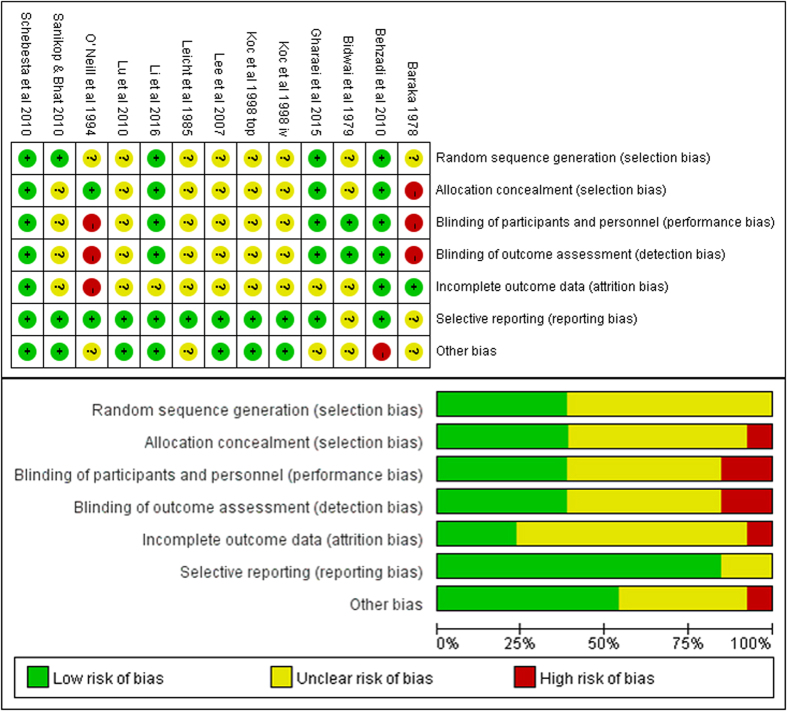
Risk of bias summary for the included studies.

The baseline characteristics were summarized in Table 1. Male/female proportion was balanced in all trials. The network of included treatment comparisons was shown in Fig. 2. The overall risk of bias of the included studies was shown in Fig. 3. Most of the included studies failed to present enough blinding detail though they declared to be double-blinded. Since the laryngospasm were judged by professional medical workers, we judged that the outcome measurement was at low risk to be influenced even when no blinding set. Predefined endpoints were reported fully in ten studies.

### Direct comparisons

**Figure 4:**
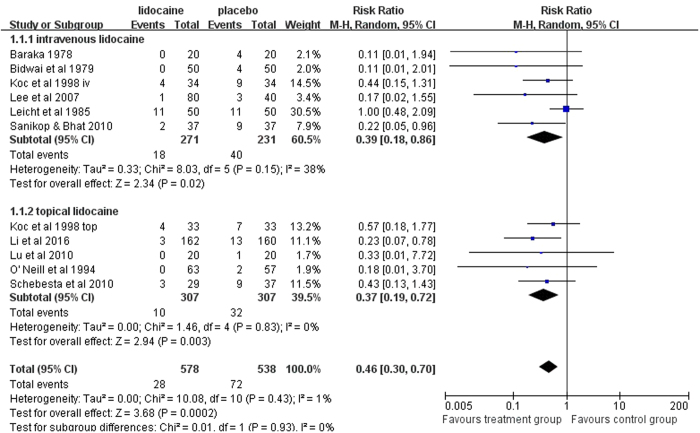
Forest plot for subgroup analysis of the efficacy of lidocaine in preventing laryngospasm in pediatric surgery.

In direct comparisons using random effect model, there were significant statistical difference in the laryngospasm incidence across the following comparisons (Fig. 4): lidocaine vs. placebo (eleven studies, 1116 participants, RR = 0.46, 95% CI = [0.30, 0.70], P = 0.0002, I^2^ = 0%); intravenous lidocaine vs. placebo (six studies, 502 participants, RR = 0.39, 95% CI = [0.18, 0.86], P = 0.02, I^2^ = 38%); topical lidocaine vs. placebo (five studies, 614 participants, RR = 0.37, 95% CI = [0.19, 0.72], P = 0.003, I^2^ = 0%). A meta-regression analysis indicated that none of the seven covariates had a significant effect (Supplementary Table S1). In intravenous lidocaine vs. topical lidocaine group (two studies, 300 participants, RR = 3.40, 95% CI = [0.07, 168.14], P = 0.54, heterogeneity P = 0.006, I^2^ = 87%), the two included studies had dramatic different results (Behzadi *et al*.^13^: RR = 23.00, 95% CI = [1.38, 384.38]; Gharaei *et al*.^12^: RR = 0.77, 95% CI = [0.46, 1.28]), thus brought high heterogeneity into the analysis and the combined result was obviously incredible.

### Network comparisons

In network comparisons of the laryngospasm incidence (Table 2), the results were similar to those in the direct comparisons. There was a great decrease in laryngospasm incidence in topical lidocaine vs. placebo group (RR = 0.14, 95% CI = [0.02, 0.55]) and intravenous lidocaine vs. placebo group (RR = 0.25, 95% CI = [0.04, 0.86]) in consistency model, while no statistical results found in intravenous lidocaine vs. topical lidocaine group (RR = 1.72, 95% CI = [0.33, 11.96]) when all the 13 studies included; a similar result was found when 2 studies excluded because of heterogeneity introduction^3^ or suspected methodology error^13^. Using the Bayesian probability analysis, we could easily concluded that topical lidocaine might have the best effect to lower the laryngospasm incidence, intravenous lidocaine the next best, and placebo the worst (Supplementary Table S2). A similar result was produced in inconsistency model (Supplementary Table S3).

### Sensitivity analysis

We changed different models for sensitivity analysis, and got similar results for the outcome in all direct comparisons. When excluding each study one by one for sensitivity, we got similar results for the outcome in all direct comparisons in fixed effect model, and in lidocaine vs. placebo group and topical lidocaine vs. placebo group in random effect model, but not in intravenous lidocaine vs. placebo group in random effect model. Among the studies included in intravenous lidocaine vs. placebo group, Leicht *et al*.^3^ got a score of three in a 7-point Modified Jadad Score^18^, and contributed most of the heterogeneity due to a dramatically different result from the others. With it excluded from intravenous lidocaine vs. placebo group, we got a risk ratio [95% CI] equal to 0.27 [0.13, 0.58] in random effect model, and similar results when excluding the rest one by one for sensitivity. Heterogeneity of direct comparisons was high in intravenous lidocaine vs. topical lidocaine group, because there were only two included studies^12^,^13^ and they had significant different results from each other, thus the combined result was incredible. No significant heterogeneity in the network comparisons was found through a node-splitting analysis (Table 3).

### Publication bias

**Figure 5:**
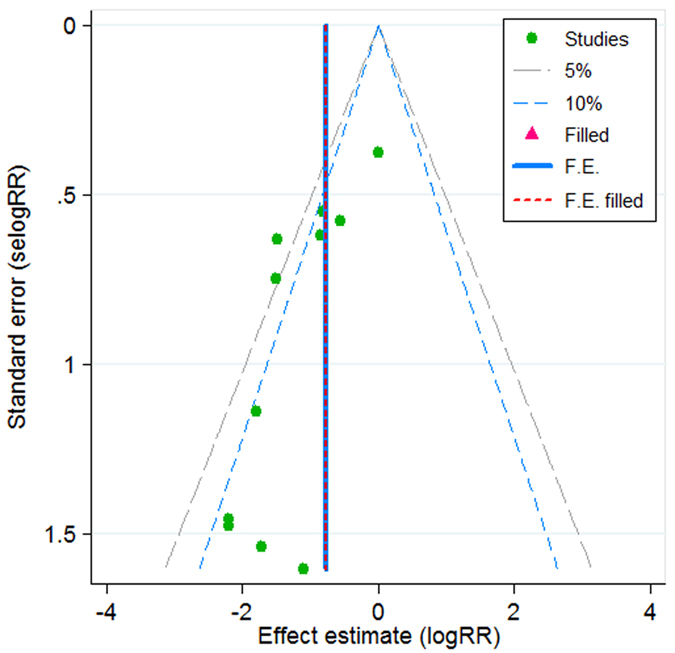
Contour-enhanced funnel plot combined with trim and fill for the publication bias of 11 trials for laryngospasm prevention in lidocaine vs. placebo group. No studies needed to be filled. The vertical solid line shows the pooled log risk ratio on the original meta-analysis, and the vertical short dashed line shows the pooled estimate including the filled studies. They overlap here since no studies needed to be filled, which indicates no publication bias.

We used a Contour-enhanced funnel plot combined with trim and fill to detect the publication bias of 11 trials in direct comparisons between lidocaine and placebo, and resulted in no studies needed to be filled (Fig. 5), suggesting no significant publication bias was found.

## Discussion

Lidocaine, either intravenously or topically, has been used for laryngospasm prevention for a long time, the effects are still inconsistent and the studies of evidence-based medicine were insufficient. We performed a standard meta-analysis and a network meta-analysis to identify the effect of lidocaine on risk of laryngospasm. In conclusion, the network comparisons and direct comparisons both indicated that both topical lidocaine and intravenous lidocaine could lower the risk of laryngospasm during general anesthesia in children, while there were no sufficient studies to compare the effect between intravenous and topical lidocaine.

In direct comparisons in lidocaine vs. placebo group, the risk ratio of laryngospasm is 0.46 [0.30, 0.70], and similar to 0.39 [0.24, 0.66] given by Mihaha *et al*.^19^. For direct comparisons in topical lidocaine vs. placebo group, the risk ratio of laryngospasm is 0.37 [0.19, 0.72], similar to 0.42 [0.22, 0.80] given by Mihaha *et al*.^19^, although we excluded one study^20^ for no full text acquired and another study^21^ for adult patients enrolled. We got a result (RR = 0.39, 95% CI = [0.18, 0.86]) similar to that (RR = 0.34, 95% CI = [0.14, 0.82]) of Mihaha *et al*.^19^ in intravenous lidocaine vs. placebo group. Different from Mihaha *et al*.^19^, we took laryngospasm events as 11 in lidocaine group and 11 in placebo group in Leicht *et al*.^3^, because the definition of laryngospasm we defined here equaled to a combination of “stridor”, “occlusion” and “cyanosis” in Leicht *et al*.^3^. Besides, we included one study in Korean^5^. And these differences during exploration and analysis contributed to the different results in sensitivity analysis. Since Leicht *et al*.^3^ was low in methodology quality according to 7-point Modified Jadad Score^18^, and contributed most of the heterogeneity due to a dramatically different result from the others in intravenous lidocaine vs. placebo group, it might be reasonable to exclude it from the meta-analysis, though a better solution was to include more high-quality studies to get a robust result.

Most studies we included are small in sample size, and the incidence of laryngospasm is low, and all these could lower the power to estimate the effect. However, Erb *et al*.^22^ conducted a clinical trial, in which they elicited respiratory reflex responses actively by spraying distilled water onto the laryngeal mucosa, so increased the incidence of laryngospasm and indicated that intravenous lidocaine had a short-lived effect (less than 10 minutes) to significantly reduce the incidence of laryngospasm in anesthetized children. In their study, the incidence of laryngospasm reduced from 38.5% to 15.4% (RR = 0.4, p = 0.011) 2 minutes after the administration of intravenous lidocaine. The risk ratio is similar to ours, which confirms our result to some extent, and reveals the need to conduct more studies for more evidence.

In Erb’s trial, the preventive effect of intravenous lidocaine was disappeared 10 minutes after administration, and most studies we included administered intravenous lidocaine within 5 minutes of extubation. These reveal that the timing of administration plays an important role in the preventive effect of intravenous lidocaine, and it might be within 5 minutes of extubation.

There are two different common methods for topical lidocaine administration, the first method is coating lidocaine gel or lubricant on the endotracheal tube or spraying lidocaine solution onto throat, thus lidocaine reacts directly and immediately on the tissue; the second is inflating the endotracheal tube cuff with lidocaine solution, thus lidocaine diffuses across the tube cuff firstly before reacting. We included 5 studies in topical lidocaine vs. placebo group, and all of them use the first method to apply topical lidocaine. Both direct and network comparisons indicated that topical lidocaine administration by the first method could lower the risk of laryngospasm. Bousselmi *et al*. conducted a trial^23^ to compare the effect of these two methods, which revealed that Intracuff lidocaine could not reduce coughing or sore throat severity in surgery of less than 120 minutes, while instilled lidocaine could. Estebe *et al*.^24^ used thin polyurethane cuff to perform an *in vitro* evaluation of diffusion of lidocaine and alkalinized lidocaine across the thin polyurethane cuff, and found that alkalinized lidocaine could increase the diffusion from <8% to >90% over a duration of 24 hours. So we suppose it is better to inflate the endotracheal tube cuff with alkalinized lidocaine solution when using the second method. In intravenous lidocaine vs. topical lidocaine group, we included a study^13^ injecting non-alkalinized lidocaine into the endotracheal tube cuff, and this could underestimate the effect of topical lidocaine. Given that we included only two studies^12^,^13^ and there was high heterogeneity in the direct comparisons between intravenous lidocaine and topical lidocaine, we believed that more studies were needed to draw a final conclusion.

Considering the timing of administration of lidocaine might also have an effect on the outcome, it was necessary to compare the effect of intravenous or topical lidocaine in trials giving lidocaine before extubation to that before intubation. However, in intravenous lidocaine vs. placebo group, all trials gave lidocaine before extubation; in topical lidocaine vs. placebo group, all trials gave lidocaine before or during intubation; in intravenous lidocaine vs. topical lidocaine group, both trials gave lidocaine before intubation, but a small sample size and high heterogeneity made the result incredible. So we were unable to evaluate the effect of intravenous or topical lidocaine according to the timing of administration, though a meta-regression analysis revealed that timing of administration didn’t affect the pooled result in the lidocaine vs. placebo group (Supplementary Table S1).

Different anesthetic gases differ in airway irritation, thus may affect the risk of laryngospasm. So we divided the trials into two groups according to the irritation of the anesthetic gases, and conducted a meta-regression analysis (Supplementary Table S1). The meta-regression analysis revealed that the irritation of the anesthetic gases didn’t affect the pooled results.

All trials judged their outcomes by skilled anesthetists, but only some of them were at low risk of bias in the blinding of outcome assessment; there were 4 studies which provided no definition of laryngospasm, and one study gave a mixed outcome containing both laryngospasm and bronchospasm. Therefore, we conducted a meta-regression analysis (Supplementary Table S1) and found that neither the blinding of outcome assessment nor the definition of laryngospasm mattered to the pooled results.

It is reported that a recent upper respiratory tract infection (URTI) within 2 weeks of the surgery and an airway procedure during the surgery could double the risk of laryngospasm in children^25^. Thus we believe it’s necessary to perform a subgroup analysis according to URTI, but we could only found one study^10^ which suggested topical lidocaine could lower the risk of laryngospasm in children with ongoing URTI or a history of recent URTI, and another study which found no efficacy difference in decreasing risk of laryngospasm in children with URTI between intravenous and topical lidocaine. And more studies are needed to conform these findings.

Undoubtedly, there are some limitations in our meta-analysis. The studies we included defined laryngospasm differently, some graded the respiratory events and provided data separately and some not, and some even provided no definition. We excluded one study^20^ because we could not get the full-text form to ensure its quality. And sensitivity analysis revealed that more studies were needed to compare the efficacy between intravenous and topical lidocaine.

In conclusion, both intravenous and topical lidocaine could prevent laryngospasm in pediatric surgery, but a comparison between them remains to be studied.

## Additional Information

**How to cite this article**: Qi, X. *et al*. The Efficacy of Lidocaine in Laryngospasm Prevention in Pediatric Surgery: a Network Meta-analysis. *Sci. Rep*. **6**, 32308; doi: 10.1038/srep32308 (2016).

## Supplementary Material

Supplementary Information

## Figures and Tables

**Table 1:** Characteristics of the included trials that assessed the efficacy of lidocaine in laryngospasm prevention in pediatric surgery.

Source	ASA physical status	Age	Surgery	Anesthetic technique	Type of airway device	Experimental group	Dose	Timing of administration	Control group	Dose	Timing of administration	URTI
Baraka^14^	NA	3–6 Ys	Tonsillectomy	Hlt(induction),Hlt (maintenance)	TT	IL	2 mg/kg	1 min before extubation	PL	NA	No intervention	NA
Bidwai *et al*.^16^	1	2–8Ys	Tonsillectomy and adenoidectomy	N2O and halothane	TT	IL	1 mg/kg	Before extubation	PL	NA	Before extubation	NA
Koc *et al*.^4^	NA	5–10 Ys	Tonsillectomy and adenoidectomy	N2O and Hlt (induction)	TT	IL	1 mg/kg	5 mins before extubation	PL	NA	5 mins before extubation	NA
Lee *et al*.^5^	1	3–10 Ys	Adenotonsillectomy	Gly, Tpt, and Vb (induction), Svf and N2O (maintenace)	TT	IL	1–2 mg/kg	1 min after beginning of spontaneous respiration	PL	NA	1 min after beginning of spontaneous respiration	No
Leicht *et al*.^3^	1	3–7 Ys	Tonsillectomy	N2O and Hlt(induction and maintenance)	TT	IL	1.5 mg/kg	3–6 min before extubation	PL	NA	3–6 min before extubation	NA
Sanikop & Bhat^9^	1, 2	3 Ms–6 Ys	Cleft palate surgeries	Ktm and Sxt(induction), N2O and Vb(maintenance)	TT	IL	1.5 mg/kg	2 min before extubation	PL	NA	2 min before extubation	No
Koc *et al*.^4^	NA	5–10 Ys	Tonsillectomy and adenoidectomy	N2O and Hlt (induction)	TT	TL	4 mg/kg	Before intubation	PL	NA	Before intubation	NA
Li *et al*.^17^	1, 2, 3	6Ms – 12Ys	Urology, otolaryngology, general, ophthalmology, orthopedic	Svf (induction), Ftn and Atc (maintenance)	TT	TL	4 mg/kg	before intubation	PL	NA	before intubation	Mixed
Lu *et al*.^15^	1,2	2 Ms–3 Ys	Cheilorrhaphy or palatorrhaphy surgery	Svf, Ftn, and Ppf, Vb(induction), Svf (maintenance)	TT	TL	1 ml of 2% lidocaine	1–2 min before intubation	PL	NA	1–2 min before intubation	NA
O’Neill *et al*.^11^	NA	4 Ms–14 Ys	NA	N2O and Hlt (induction), N2O and Hlt or Ifu (maintenance)	LMA	TL	Approximately 1/4 teaspoon of 2% viscous lidocaine	During insertion	PL	NA	During insertion	NA
Schebesta *et al*.^10^	1,2	1–10 Ys	Minor surgical procedures	Svf, Ftn, and Ppf (induction), Ftn and Svf (maintenance)	LMA	TL	0.3 ml/kg	During insertion	PL	NA	During insertion	Subgroup
Gharaei *et al*.^12^	NA	1–6 Ys	Full ophthalmic examination	Svf(induction), N2O, Svf (maintenance)	LMA	IL	1.5 mg/kg	Before anesthesia	TL	0.1 mL/kg of of 2% lidocaine	During insertion	Yes
Behzadi *et al*.^13^	1, 2	5–10 Ys	Adenotonsillectomy	Mdz and Ftn,sodium Tpt and Atc(induction), N2O, Ifu(maintenance)	TT	IL	1.5 mg/kg	Immediately after intubation	TL	A maximum dosage of 5 mg/kg of 2% lidocaine	Immediately after intubation	No

Abbreviations: NA, missing data; TT, tracheal tube; LMA, laryngeal mask airway; TL, topical lidocaine; IL, intravenous lidocaine; PL, placebo; URTI, upper resperatory tract infection; Hlt, halothane; Svf, sevoflurane; Ifu, isoflurane; Ftn, fentanyl; Ppf, propofol; Vb, vecuronium bromide; Gly, glycopyrrolate; Tpt, thiopental; Ktm, ketamine; Sxt, suxamethonium; Mdz, Midazolam; Atc, atracurium.

**Table 2:** The effects of the laryngospasm interventions on the laryngospasm incidence in consistency model.

Intravenous lidocaine	1.72 (0.33, 11.96)	**0.25 (0.04, 0.86)**
0.63 (0.22, 1.60)	Topical lidocaine	**0.14 (0.02, 0.55)**
**0.17 (0.05, 0.35)**	**0.27 (0.10, 0.64)**	Placebo

Data was listed as RR with 95% CI. Effect estimates from the network meta-analysis including all the 13 studies in the consistency model occupy the top right part of the diagram, and the estimates with 2 studies excluded occupy the bottom left part of the diagram. The diagonal corresponds to the comparison. The diagonal corresponds to the comparison. Significant results are in bold. The data should be read from left to right.

**Table 3:** Node-splitting analysis of inconsistency in the network comparisons.

Name	Direct Effect	Indirect Effect	Overall	P-Value
IL, PL	1.78 (0.21, 3.95)	0.23 (−3.85, 3.62)	1.39 (0.15, 3.16)	0.36
IL, TL	−1.40 (−5.08, 1.16)	0.12 (−2.61, 2.88)	−0.54 (−2.48, 1.11)	0.36
PL, TL	−1.65 (−4.06, 0.16)	−3.15 (−7.64, −0.26)	−1.94 (−4.03, −0.60)	0.38

P < 0.05 means high heterogeneity in that comparison. Abbreviations: IL, intravenous lidocaine; TL, topical lidocaine; PL, placebo.

## References

[b1] Olsson G. L. & Hallen B. Laryngospasm during anaesthesia. A computer-aided incidence study in 136,929 patients. Acta Anaesthesiol Scand 28, 567–575 (1984). 6496018 10.1111/j.1399-6576.1984.tb02121.x

[b2] Tay C. L, Tan G. M. & Ng S. B. Critical incidents in paediatric anaesthesia: an audit of 10 000 anaesthetics in singapore. Paediatr Anaesth 11, 711–718 (2001). 11696149 10.1046/j.1460-9592.2001.00767.x

[b3] Leicht P., Wisborg T. & Chraemmer-Jorgensen B. Does intravenous lidocaine prevent laryngospasm after extubation in children? Anesth Analg 64, 1193–1196 (1985). 4061902

[b4] Koc C., Kocaman F., Aygenc E., Ozdem C. & Cekic A. The use of preoperative lidocaine to prevent stridor and laryngospasm after tonsillectomy and adenoidectomy. Otolaryngol Head Neck Surg 118, 880–882 (1998). 9627258 10.1016/S0194-5998(98)70290-6

[b5] Lee J. Y., Kim C. H., Kim S. H., Kim J. & Lee K. Intravenous lidocaine prior to extubation reduces emergence agitation and cough in pediatric adenotonsillectomy under sevoflurane anesthesia. Korean Journal of Anesthesiology 53, 458–462 (2007).

[b6] Hamilton N. D., Hegarty M., Calder A., Erb T. O. & von Ungern-Sternberg B. S. Does topical lidocaine before tracheal intubation attenuate airway responses in children? An observational audit. Paediatr Anaesth 22, 345–350, doi: 10.1111/j.1460-9592.2011.03772.x (2012). 22211867

[b7] Higgins J. P. & Green S. Cochrane handbook for systematic reviews of interventions version 5.1.0. (2011) Available at: http://handbook.cochrane.org/. (Accessed:18th October 2015).

[b8] Palmer T. M., Peters J. L., Sutton A. J. & Moreno S. G. Contour-enhanced funnel plots for meta-analysis. Stata J 8, 242–254 (2008).

[b9] Bhat S. & Sanikop C. S. Efficacy of intravenous lidocaine in prevention of post extubation laryngospasm in children undergoing cleft palate surgeries. Indian Journal of Anaesthesia 54, 132, doi: 10.4103/0019-5049.63654 (2010). 20661351 PMC2900736

[b10] Schebesta K., Güloglu E., Chiari A., Mayer N. & Kimberger O. Topical lidocaine reduces the risk of perioperative airway complications in children with upper respiratory tract infections. Canadian Journal of Anesthesia/Journal canadien d’anesthésie 57, 745–750, doi: 10.1007/s12630-010-9328-y (2010). 20524104

[b11] O’Neill B., Templeton J. J., Caramico L. & Schreiner M. S. The laryngeal mask airway in pediatric patients: factors affecting ease of use during insertion and emergence. Anesth Analg 78, 659–662 (1994). 8135383

[b12] Gharaei B. . Topical versus intravenous lidocaine in children with upper respiratory infection undergoing anesthesia: a randomized, double blind, clinical trial. Anesthesiology and Pain Medicine 5, e23501, doi: 10.5812/aapm.23501v2 (2015). 26473098 PMC4602159

[b13] Behzadi M., Hajimohamadi F., Alagha A. E., Abouzari M. & Rashidi A. Endotracheal tube cuff lidocaine is not superior to intravenous lidocaine in short pediatric surgeries. Int J Pediatr Otorhi 74, 486–488, doi: 10.1016/j.ijporl.2010.01.025 (2010). 20189659

[b14] Baraka A. Intravenous lidocaine controls extubation laryngospasm in children. Anesth Analg 57, 506–507 (1978). 360878 10.1213/00000539-197807000-00028

[b15] Lu D., Cheng D. & Xiong L. Therapeutic efficacy of different polydocanols on complications of endotracheal intubation in infants. The Journal of Clinical Anesthestology 26, 497–499 (2010).

[b16] Bidwai A. V., Rogers C. & Stanley T. H. Prevention of post-extubation laryngospasm after tonsilectomy. Anesthesiology 51 (1979).

[b17] Li L., He L., Ai Y., Chu Q. & Zhang W. Site-directed topical lidocaine spray attenuates perioperative respiratory adverse. J Surg Res (2016). 10.1016/j.jss.2016.03.011 27338551

[b18] Jadad A. R. . Assessing the quality of reports of randomized clinical trials: is blinding necessary? Control Clin Trials 17, 1–12 (1996). 8721797 10.1016/0197-2456(95)00134-4

[b19] Mihara T., Uchimoto K., Morita S. & Goto T. The efficacy of lidocaine to prevent laryngospasm in children: a systematic review and meta-analysis. Anaesthesia 69, 1388–1396, doi: 10.1111/anae.12788 (2014). 24992191

[b20] Penaloza I., Diaz M. & Jimenez T. Use of beclametazone dipropionate for prevention of post-intubation laryngospasm in pediatrics vs topical lidocaine. Anestesia en Mexico (1999).

[b21] Staffel J. G., Weissler M. C., Tyler E. P. & Drake A. F. The prevention of postoperative stridor and laryngospasm with topical lidocaine. Arch Otolaryngol Head Neck Surg 117, 1123–1128 (1991). 1910697 10.1001/archotol.1991.01870220071012

[b22] Erb T. O., Von Ungern-Sternberg B. S., Keller K. & Frei F. J. The effect of intravenous lidocaine on laryngeal and respiratory reflex responses in anaesthetised children. Anaesthesia 68, 13–20, doi: 10.1111/j.1365-2044.2012.07295.x (2013). 23061716

[b23] Bousselmi R. . Lidocaine reduces endotracheal tube associated side effects when instilled over the glottis but not when used to inflate the cuff: a double blind, placebo-controlled, randomized trial. Tunis Med 92, 29–33 (2014). 24879167

[b24] Estebe J. P. . *In vitro* evaluation of diffusion of lidocaine and alkalinized lidocaine through the polyurethane membrane of the endotracheal tube. Annales Françaises d’Anesthésie et de Réanimation 33, e73–e77, doi: 10.1016/j.annfar.2013.12.022 (2014). 24582110

[b25] Schreiner M. S., O’Hara I., Markakis D. A. & Politis G. D. Do children who experience laryngospasm have an increased risk of upper respiratory tract infection? Anesthesiology 85, 475–480 (1996). 8853076 10.1097/00000542-199609000-00005

