# Supplementary material for: The Efficacy of Lidocaine in Laryngospasm Prevention in Pediatric Surgery: a Network Meta-analysis
Source: Sci Rep. 2016 Sep 2;6:32308. doi: 10.1038/srep32308 (PMC5009364; doi:10.1038/srep32308)
Supplement: Supplementary Information [file srep32308-s1.pdf]

Supplementary Material: the Efficacy of Lidocaine in Laryngospasm Prevention in Pediatric Surgery: a Network Meta-analysis

Xiaojing Qi[1,#], Zhoupeng Lai[1,#], Si Li[1], Xiaochen Liu[1], Zhongxing Wang[2,*], Wulin Tan[2,*]

[1]Zhongshan School of Medicine, Sun Yat-sen University, Guangzhou 510080, China.

[2]Department of Anesthesiology, The First Affiliated Hospital, Sun Yat-sen University, Guangzhou 510080, China.

[*]Zhongxing Wang (Email: doctorwzx@126.com) and Wulin Tan (Email: tanwulin1986@163.com) are the corresponding authors.

[#]These authors contributed equally to this work and should be considered co-first authors.

Correspondence and requests for materials should be addressed to Zhoupeng Lai (Email: laizhoup@mail2.sysu.edu.cn) or Zhongxing Wang (Email: doctorwzx@126.com).

Table S1: Results of meta-regression analysis to determine whether covariates had a significant effect.

**Covariate**	**P value**	
	Univariate meta-regression	Multivariate meta-regression
**Surgery**	0.152	0.262
**Anesthetic gas**	0.617	0.542
**Airway device**	0.886	0.209
**Route of administration**	0.762	0.385
**Timing of administration**	0.762	CN
**Definition of laryngospasm**	0.306/CN/ 0.877	0.167/CN/ 0.254
**Blinding of outcome assessment**	0.362	0.269

P＜0.05 means significant. Abbreviations: CN, collinearity.

Table S2: Probability for each alternative to be at each rank given the analysis model and data.

**Drug**	**Rank1**	**Rank2**	**Rank3**
**Intravenous lidocaine**	0.02	0.75	0.23
**Placebo**	0.98	0.02	0.00
**Topical lidocaine**	0.00	0.23	0.77

Rank 1 is worst, Rank 3 is best. The bigger number in the rank, the higher probability to be better in that rank.

Table S3: The effects of the laryngospasm interventions on the laryngospasm incidence in inconsistency model.

Intravenous lidocaine	2.33 (0.33, 27.75)	**0.22 (0.04, 0.84)**
0.65 (0.18, 2.23)	Topical lidocaine	**0.16 (0.02, 0.75)**
**0.16 (0.05, 0.39)**	**0.26 (0.08, 0.61)**	Placebo

Data was listed as RR with 95% CI. Effect estimates from the network meta-analysis including all the 13 studies in the inconsistency model occupy the top right part of the diagram, and the estimates with 2 studies excluded occupy the bottom left part of the diagram. The diagonal corresponds to the comparison. Significant results are in bold. The data should be read from left to right.
